# Successful Treatment of Pediatric Refractory/Relapsed AML with KIR-Ligand-Mismatched Cord Blood Transplant after FLAG-IDA Reinduction Therapy with or without the GO Regimen

**DOI:** 10.1155/2020/1378056

**Published:** 2020-02-12

**Authors:** Daisuke Toyama, Ryosuke Matsuno, Yumiko Sugishita, Ryota Kaneko, Naoko Okamoto, Masaya Koganesawa, Sachio Fujita, Kosuke Akiyama, Keiichi Isoyama, Shohei Yamamoto

**Affiliations:** Department of Pediatrics, Showa University Fujigaoka Hospital, Yokohama, Japan

## Abstract

Prognosis in pediatric patients with refractory/relapsed acute myeloid leukemia (AML) is grim, and there is no standard treatment for such patients. Combined treatment with intensive chemotherapy and gemtuzumab ozogamicin (GO), a monoclonal anti-CD33 antibody conjugated with calicheamicin, is useful as reinduction therapy in refractory/relapsed AML. Here, we describe three cases of pediatric refractory/relapsed AML that were successfully managed with FLAG-IDA (fludarabine, cytarabine, granulocyte colony-stimulating factor, and idarubicin), with or without GO, as reinduction therapy before a KIR-ligand-mismatched cord blood transplant. This strategy relies on the fact that killer cell immunoglobulin-like receptors (KIR) on cord blood natural killer (NK) cells recognize human leukocyte antigen (HLA) class I alleles, and that donor KIR-ligand incompatibility may be associated with lower incidence of relapse and improved survival in AML, as cells that lack these inhibitory HLA ligands can activate NK cells. All three patients are currently alive and have been disease-free for 24–65 months, although one patient developed severe sinusoidal obstructive syndrome (SOS). Thus, our strategy can result in excellent outcomes in pediatric patients with refractory/relapsed AML.

## 1. Introduction

Outcomes in children with refractory/relapsed acute myeloid leukemia (AML) are extremely poor [1]. Fludarabine, cytarabine, granulocyte colony-stimulating factor (G-CSF), and idarubicin (FLAG-IDA) is a commonly used reinduction regimen in AML patients [2], and FLAG-IDA combined with gemtuzumab ozogamicin (GO) is a feasible reinduction regimen for relapsed AML [3, 4]. Killer cell immunoglobulin-like receptor (KIR)-ligand incompatibility is thought to be associated with reduced incidence of relapse and better leukemia-free survival [5, 6] because clinically beneficial graft versus leukemia effects might be mediated by donor-recipient inhibitory KIR-mismatched natural killer (NK) cells [7]. Here, we describe the cases of three patients with pediatric refractory/relapsed AML who were treated using the FLAG-IDAregimen, with or without GO, as a reinduction therapy before KIR-ligand-mismatched cord blood transplant (KIR-CBT).

## 2. Case Presentations

Between April 2011 and December 2015, one male and two female pediatric patients with relapsed or refractory AML were treated with one course of FLAG-IDA, with or without GO, after which they underwent KIR-CBT. Patient characteristics are listed in Table 1. All three patients displayed one or more poor prognostic factors, as identified by previous studies [1, 8]. One patient (case 1) suffered from refractory (induction failure) AML, while the other two (cases 2 and 3) had relapsed AML. Among the 2 cases of relapsed AML, case 2 underwent HSCT after the first complete remission (1 CR) because she had a high-risk karyotype (5q-), while case 3 suffered an early relapse. Cases 1 and 3 were administered FLAG-IDA with GO as reinduction therapy prior to KIR-CBT, while case 2 was given only FLAG-IDA as only 18% of her leukemic cells expressed CD33. The FLAG-IDA protocol comprised 30 mg/m^2^ fludarabine and 2 g/m^2^ cytarabine on days 2–6, 10 mg/m^2^ idarubicin on day 6, and 300 *µ*g/m^2^ G-CSF on days 1–6. GO was administered at a dose of 3 mg/m^2^ on day 7 of treatment, and its use was approved by the institutional review board of the Showa University Fujigaoka Hospital, Japan. All cases achieved CR after reinduction therapy (Table 1) and promptly underwent KIR-CBT (Table 2). KIR-ligand mismatching was defined based on KIR-ligand incompatibility, as initially described by Leung [9]. All three patient-donor pairs were KIR-ligand-incompatible in the graft-versus-host (GVH) direction and were all donor-mismatched for KIR2DL1 with HLA-C missing from the recipient. Cases 1 and 3 received full-intensity conditioning cyclophosphamide (120 mg/kg) and total body irradiation (TBI: 12 Gy), while case 2 received melphalan (140 mg/m^2^), fludarabine (125 mg/m^2^), and TBI (2 Gy), as this was her 2^nd^ HSCT. Anti-thymocyte globulin (ATG) was not used in any of the patients, and tacrolimus (0.02 mg/kg/day) and short-term methotrexate were used for prophylaxis against graft-versus-host disease (GVHD). Ursodeoxycholic acid (UDCA) and low-molecular-weight heparin (LMWH) were administered for prophylaxis against sinusoidal obstruction syndrome (SOS) in case 1, while cases 2 and 3 received prophylactic recombinant human soluble thrombomodulin (rTM; 380 U/kg per day for 7 days from days 7 to 13), in addition to UDCA and LMWH [10]. All three cases underwent KIR-CBT within 2 months of reinduction therapy. In case 1, extremely severe SOS developed on day 8 which was treated with rTM; however, this was ineffective, and continuous hemodiafiltration was initiated to maintain hydration and remove any inflammatory cytokines. This resulted in a gradual improvement in her SOS by day 20. Additionally, Grade I acute GVHD (stage 2 skin rash) and limited chronic GVHD (affecting the skin alone) developed in this patient, which were successfully treated with prednisolone (PSL). Tacrolimus was suspended at 9 months after KIR-CBT. In cases 2 and 3, prophylactic rTM was successful and they did not develop SOS. In case 2, no acute or chronic GVHD occurred, and the administration of tacrolimus was stopped at 5 months after KIR-CBT. In case 3, grade III acute GVHD (stage 3 skin rash and stage 3 diarrhea) developed, and it responded well to PSL treatment. Chronic GVHD did not occur in this patient, and the administration of tacrolimus was stopped at 5 months after KIR-CBT. All three patients are currently alive without any evidence of relapse at 24–65 months after KIR-CBT.

## 3. Discussion

Prognosis in pediatric patients with refractory/relapsed AML is poor at best and known prognostic factors for poor survival include early relapse, undergoing HSCT during 1 CR, age of >10 years at relapse, and adverse cytogenetics [1]. Since April 2011, our strategy for treating patients with refractory or relapsed AML having poor prognostic factors has been one course of FLAG-IDA, with or without GO, followed by KIR-CBT. This strategy has two major implications for the treatment of pediatric patients with refractory/relapsed AML, especially those with poor prognostic factors. First, the FLAG-IDA regimen, with or without GO, might be a feasible reinduction therapy. This is because, among pediatric patients with relapsed AML, those who do not achieve 2 CR exhibit significantly lower 5-year OS rate than the patients who achieve 2 CR [1, 8]. A European study group has reported that FLAG, with or without an anthracycline, can induce 2 CR in 78% of the cases, which is an excellent rate of induction [1]. GO, a monoclonal anti-CD33 antibody conjugated with calicheamicin, has been shown to be effective in relapsed AML [11], and FLAG-IDA, combined with low-dose GO (3 mg/m^2^), is a feasible induction therapy option in younger patients with primary AML [3, 4]. All three of our patients also achieved CR after being treated with FLAG-IDA, with or without GO. Second, we used KIR-CBT (a type of HSCT) in pediatric refractory/relapsed AML patients with poor prognostic factors because of the following rationale. NK cells express inhibitory KIR on their surface, and NK cell alloreactivity after allogeneic transplantation is determined by the specificity of the KIR on the donor NK cells for the recipient's MHC (major histocompatibility complex) class I antigens [12]. Ruggeri et al. were the first to report good clinical results, i.e., no relapse, rejection, or acute GVHD in AML patients who underwent HLA-mismatched transplantation involving KIR-ligand incompatibility in the GVH direction [5]. In a study wherein approximately 80% of the patients were administered ATG, Willemze et al. have reported that KIR-ligand incompatibility had a beneficial effect on outcomes after CBT [13]. In contrast, in a study in which only 30% of the patients were administered ATG, Brunstein et al. have reported that KIR-ligand incompatibility had no effect on outcomes after CBT [14]. It is possible that in vivo T-cell depletion, secondary to ATG administration, contributed to the posttransplant expansion of functional NK cells which then favored alloreactivity in the presence of KIR-ligand mismatching. Although none of our patients were given ATG, there were no relapses, and acute GVHD remained under control. This may be because many Japanese express HLA-C1 and few episodes of acute GVHD have been recorded after KIR-ligand-mismatched HSCT in Japan [15]. Rubnitz et al. have reported no relapses in pediatric AML patients who underwent KIR-ligand-mismatched HSCT after achieving CR [16]. As these results suggest that NK cell alloreactivity affects the extent of minimal residual disease, KIR-CBT should be performed as soon as possible after CR has been achieved. Although all three of our patients successfully achieved CR, evidence for the efficacy of including GO during induction therapy and KIR-CBT in pediatric AML remains inconclusive. Recent advances in AML treatment have identified several promising modalities, such as antimethylating agents, immune checkpoint inhibitors (ICIs), and chimeric antigen receptor-T cells (CAR-T). Daver et al. have reported that Azacitidine, in combination with ICIs, may be safe and effective in patients with relapsed AML [17], while Li et al. have stated that CD33 CAR-T is effective against AML cells and that it prolongs survival in a mouse xenograft model [18]. Those new drugs can alter the treatment landscape of pediatric refractory/relapsed AML and provide new treatment opportunities in the future.

In conclusion, the FLAG-IDA reinduction therapy described here, with or without GO, when used before KIR-CBT, appears to be a feasible treatment strategy in pediatric patients with refractory/relapsed AML. However, definitive conclusions about the efficacy of this approach cannot be made because of the very small number of patients included in this study. Therefore, additional prospective studies are required to evaluate the same.

## Figures and Tables

**Table 1:** Characteristics of the three patients with pediatric refractory/relapsed acute myeloid leukemia.

	Case 1	Case 2	Case 3
Age of induction failure or relapse/gender	2 years/female	18 years/female	13 years/male
Diagnosis (FAB classification)	M2	M0	M2
CD33 expression level (%)	90	18	84
Karyotype	46, XX	46, XX. Del (5)	46, XY
FLT3-ITD	None	None	None
Disease status	Induction failure	1st relapse	1st relapse
Poor prognostic factor(s)			
Induction failure	Yes	No	No
Age >10 years at relapse	No	Yes	Yes
Early relapse (1 year from diagnosis)	—	No	Yes (11 months)
HSCT at 1st CR	No	Yes	No
Adverse cytogenetics	No	Yes	No

FAB, French-British-American classification; HSCT, hematopoietic stem-cell transplantation; 1 CR, first complete remission; 2 CR, second complete remission, FLAG, fludarabine, cytarabine, granulocyte colony-stimulating factor; IDA, idarubicin; GO, gemtuzumab ozogamicin.

**Table 2:** Characteristics of KIR-CBT in the three patients.

	Case 1	Case 2	Case 3
Disease status at HSCT	1 CR	2 CR	2 CR
Interval between GO and HSCT (days)	36	ー	56
Conditioning	TBI + CY	LPAM + Flu + TBI2Gy	TBI + CY
Donor source	UR-CB	UR-CB	UR-CB
HLA match (DNA typing)	5/8 (B, C, DR)	5/8 (B, C)	5/8 (B, C, DR)
HLA-C			
Patient	C08 : 01/12 : 03 (C1/C1)	C03 : 04/14 : 02 (C1/C1)	C03 : 03/07 : 02 (C1/C1)
Donor	C08 : 01/04 : 01 (C1/C2)	C01 : 02/04 : 01 (C1/C2)	C04 : 01/07 : 02 (C1/C2)
KIR-HLA			
GVH direction mismatch	KIR2DL1 (inhibitory KIR)	KIR2DL1 (inhibitory KIR)	KIR2DL1 (inhibitory KIR)
NCC (×10^7^/kg)	6.1	3.2	2
Engraftment (Neutro > 0.5 × 10^9^/L)	Day 36	Day 32	Day 30
SOS prophylaxis	Ursodeoxycholic acid + LMWH	Ursodeoxycholic acid + LMWH + rTM	Ursodeoxycholic acid + LMWH + rTM
SOS	Very severe	—	—
GVHD			
GVHD prophylaxis	Tacrolimus + MTX	Tacrolimus + MTX	Tacrolimus + MTX
aGVHD	Grade I (skin: stage 2)	—	Grade III (skin: stage 3, gut: stage 3)
cGVHD	Limited (skin)	—	—
Treatment of aGVHD	PSL	—	PSL
Infection	—	CMV viremia	—
Prognosis	Alive (65 months)	Alive (34 months)	Alive (24 months)

KIR-CBT, KIR-ligand-mismatched cord blood transplant; HSCT, hematopoietic stem-cell transplantation; GO, gemtuzumab ozogamicin; HLA, human leukocyte antigen; GVH, graft-versus-host; NCC, nucleated cord blood cells; SOS, sinusoidal obstruction syndrome; GVHD, graft-versus-host disease; aGVHD, acute GVHD, cGVHD, chronic GVHD; CR, complete remission; TBI, total body irradiation; CY, cyclophosphamide; LPAM, melphalan; Flu, fludarabine; UR, unrelated; CB, cord blood; rTM, recombinant human soluble thrombomodulin: LMWH, low-molecular-weight heparin; tacrolimus; MTX, methotrexate; PSL, prednisolone; CMV, cytogenesis virus.
